# Electrowinning of Nickel from Lithium-Ion Batteries

**DOI:** 10.3390/ma18245653

**Published:** 2025-12-16

**Authors:** Katarzyna Łacinnik, Szymon Wojciechowski, Wojciech Mikołajczak, Artur Maciej, Wojciech Simka

**Affiliations:** 1 Department of Inorganic Chemistry, Analytical Chemistry, and Electrochemistry, Faculty of Chemistry, Silesian University of Technology, 44-100 Gliwice, Poland; katarzyna.pieta@polsl.pl (K.Ł.); artur.maciej@polsl.pl (A.M.); 2 Elion Sp. z o.o., 05-119 Legionowo, Poland; szymon.wojciechowski@elion-circular.com (S.W.); wojciech.mikolajczak@elion-circular.com (W.M.)

**Keywords:** nickel, electrowinning, lithium-ion batteries, recovery

## Abstract

The growing demand for lithium-ion batteries (LIBs) is driving a rapid increase in the volume of spent cells which—as hazardous waste—must be managed effectively in accordance with circular-economy principles. Hydrometallurgical recycling allows the recovery of critical metals at far lower environmental cost than primary mining. This paper presents a method for obtaining metallic nickel from sulfate leach solutions produced by leaching the so-called “black mass” derived from shredded LIBs. Nickel electrodeposition was performed on a stainless-steel cathode with Ti/Ru-Ir anodes at 60 °C and pH 3.0–4.5. Two process variants were examined. Variant A—with a decreasing Ni^2+^ concentration (49 → 25 g L^−1^)—achieved a current efficiency of 60–88%, but the deposits were non-uniform and prone to flaking. Variant B—in which the bath was stabilized by the continuous dissolution of Ni(OH)_2_ (maintaining Ni^2+^ at 35–40 g L^−1^) and amended with PEG-4000, H_3_BO_3_ and Na_2_SO_4_—reached higher efficiency (78–93%) and produced uniform, bright deposits up to 0.5 mm thick with a purity >90%. The results confirm that keeping the nickel concentration constant and appropriately modifying the electrolyte significantly improve both the qualitative and economic aspects of recovery, highlighting electrolysis as an efficient way to process LIB waste and close the nickel stream within the material cycle.

## 1. Introduction

The growing demand for lithium-ion batteries (LIBs)—used in electric vehicles and stationary energy-storage systems—is sharply increasing the need for critical metals such as lithium, cobalt, nickel and manganese [1]. These raw materials have been classified as critical to the security of supply for many economies. At the same time, extracting them from ores entails considerable environmental and social costs [2], while obtaining lithium from brines consumes significant amounts of water (around 2 million litres per tonne of Li) [3]. Lithium-ion battery recycling is therefore seen as a key element of the circular economy that can secure metal supplies and reduce environmental impacts [2].

Lithium-ion battery recycling employs several competing technologies, each with clear strengths and limitations. Pyrometallurgy remains the most mature and industrially adopted method thanks to its simple process flow and minimal need for sorting, though it recovers only a limited range of metals and suffers from high energy demand, toxic emissions, and associated environmental costs [4]. Hydrometallurgy enables the high-purity recovery of nearly all battery components with lower energy use but relies on complex chemical steps and strong inorganic acids that generate hazardous gases, solid waste, and acidified effluents. Organic-acid leaching offers greener pathways but still faces slow kinetics and higher costs. Direct recycling shows strong potential for economic and environmental benefits because of its low-temperature, low-energy processing, yet remains constrained by strict feedstock requirements, extensive pre-sorting, and its early-stage technological maturity [5]. Bioleaching offers a low-cost and environmentally benign alternative using microorganisms to dissolve metals, but its slow reaction rates and susceptibility to contamination hinder industrial deployment. Electrometallurgy provides fast, selective metal recovery with high purity and tunable process conditions, though its economic and environmental performance depends heavily on electricity costs and the availability of renewable power. Together, these approaches illustrate a rapidly evolving landscape in which no single method is universally optimal, and future progress will depend on integrating technologies, improving sustainability assessments, and adapting processes to the changing composition of next-generation LIBs [6].

Hydrometallurgical processing of black mass can yield a variety of products, including purified metal salts (e.g., sulfates, chlorides or carbonates), hydroxides and metal oxides, and—when suitably integrated separation and refining stages are used—metals in elemental form (e.g., cobalt, nickel). One of the most promising methods of recovering nickel from battery material is its electrolytic deposition, which provides high-purity metal that can be re-introduced into the economic cycle.

The first stage of lithium-ion battery recycling is mechanical pre-treatment, which involves shredding and mechanical separation of the resulting fractions to obtain so-called black mass—a mixture of graphite or silicon and cathode material. A second solid stream produced during milling is the coarse fraction, a mixture of steel scrap, copper, aluminium and plastic foils. A lithium-ion battery also contains a liquid electrolyte—usually one or more carbonates (dimethyl, diethyl, ethyl–methyl, ethylene, propylene). The salt LiPF_6_ is also commonly added [7]. Because of the presence of organic solvents, some processes include vacuum drying or roasting of the black mass before hydrometallurgical treatment [8]. Hydrometallurgical methods recover valuable elements from black mass with chemical reagents at much lower temperatures than pyrometallurgical routes [6]. At present, hydrometallurgy is considered the most effective and relatively energy-efficient industrial method for recovering metals from NMC cathodes [2].

Acid leaching of black mass—that is mix of the active cathode and anode materials—yields solutions with a complex, variable, multi-element composition, among other factors, it depends strongly on the battery chemistry (e.g., the Ni:Mn:Co ratio in an NMC cathode or the presence of other metals in NCA or LFP cathodes) and on the leaching parameters themselves (acid type and concentration, temperature, time, liquid-to-solid ratio, type of a reductant) [9]. Leaching is commonly carried out with 1–3 M sulfuric acid with a reductant (H_2_O_2_, SO_2_) at 60–90 °C for 1–3 h. The liquid-to-solid ratio is usually 5:1 to 10:1 [10].

In the recycling of lithium-ion batteries, the hydrometallurgical solutions used are completely different from those applied to NiMH cells, because they dissolve lithium, cobalt, nickel and manganese—typically using sulfuric acid with hydrogen peroxide—whereas NiMH batteries are leached mainly in sulfuric or hydrochloric acid to recover nickel [11] and rare-earth elements such as lanthanum and cerium [12]. Nickel–cadmium batteries, on the other hand, are processed primarily through pyrometallurgy, where high temperatures allow cadmium to evaporate and condense as a purified metal, eliminating the need for complex leaching solutions. As a result, the hydrometallurgical chemistry used for Li-ion batteries is more complex and focused on recovering strategic metals, while NiMH and NiCd batteries require simpler (NiMH) or entirely different (NiCd) treatment methods [13].

Consequently, the solutions contain ions of valuable metals such as Ni, Co, Li and Mn, but also a series of metals and impurities at lower concentrations (e.g., Al, Fe, Cu, Mg, Zn) [14,15]. The presence of many species with often similar chemical properties makes the efficient separation of individual metals a major industrial challenge. The variability in leach-solution composition therefore demands flexible and selective separation methods, which typically translates into complex multi-stage flowsheets [16] (precipitation, solvent extraction [17], ion exchange or electrolysis). The choice of the optimal sequence of operations depends both on the desired final product [18] and on the technological and economic constraints of the recycling plant.

For nickel recovery, it is crucial to reduce the concentration of impurities that interfere with electrolysis. Iron, aluminum or manganese ions lower the efficiency of nickel deposition and the purity of the product. Appropriate post-leach solution treatment aimed at selectively removing—or at least lowering the concentration of—unwanted metals is therefore essential before the main nickel-recovery stage, whether it proceeds by hydrometallurgical or electrochemical means.

Recent industrial-scale hydrometallurgical flowsheets—such as Recupyl/TES-AMM, LithoRec (Volkswagen), Aalto University, Lithion Recycling, Li-Cycle and American Technology Company—share a common feature: nickel is typically recovered as an intermediate compound (NiSO_4_, NiCO_3_ or Ni(OH)_2_), whereas metallic nickel is not produced directly from the process solutions. In most commercial operations, electrowinning is applied only to cobalt (e.g., Recupyl and Lithion), while nickel is routed back to precursor or sulfate production steps [2].

A major reason for this limitation is the variable and impurity-rich composition of leachates generated during mechanical pre-treatment, impurity removal and solvent extraction. These fluctuations make it difficult to maintain the stable electrochemical conditions required for high-quality nickel deposition, particularly when thick (>300–400 μm) metallic layers are needed.

Methods for removing impurities may be chosen to minimize losses of valuable elements. In a multi-stage process that generates many side streams, limiting the loss of precious metals is particularly important. Producing higher-purity products is often accompanied by a decrease in overall recovery: the more aggressive or selective the separation step, the greater the risk that some Ni, Co or Li ions will end up in waste streams along with the removed impurities. In practice, this means continually balancing two opposing goals: meeting purity specifications (e.g., ≥99.9% for battery-grade cathode material) and maintaining high overall metal recoveries (≥90%) [19].

**Figure 1:**
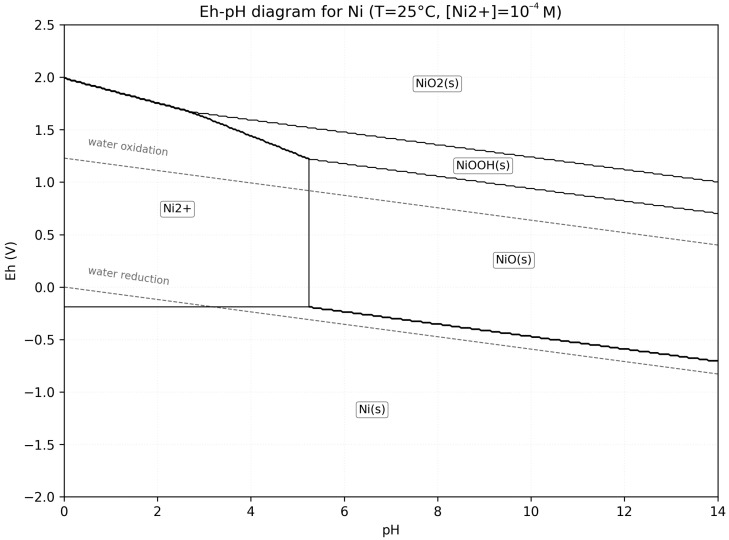
Electrochemical stability diagram of nickel: predominant aqueous and solid phases vs. pH and Eh (25 °C, 10^−4^ M Ni^2+^) [21,22].

Holm investigated process parameters in nickel electrolysis from sulfate solutions include, bath composition, temperature, pH and current density [20]. Nickel sulfate (NiSO_4_), was used at 100–150 g L^−1^. Raising the temperature from 40 °C to 60 °C influence nickel deposition and yields coatings with lower stress and more uniform structure. The pH was kept between 2.0 and 3.5 Raising electrolyte temperature from 40 °C to 60 °C improves deposit morphology and lifts current efficiency. Increasing pH from 2.0 to 3.5 boosts efficiency but introduces cracking, curling, and oxide/hydroxide inclusions; higher temperature mitigates some of these defects. Elevating Ni^2+^ concentration further enhances quality and efficiency, though its influence is weaker than that of pH or temperature. Temperature remains the primary control lever, but all three parameters must stay within optimal limits to obtain sound nickel cathodes [20]. As indicated by the Pourbaix diagram (Figure 1), lower pH-values promote hydrogen evolution at the cathode, reducing efficiency, whereas values that are too high cause nickel oxide or nickel hydroxy oxide precipitation.

Another crucial parameter is Limiting current density [23]. It’s the maximum current density at which the process can proceed without depleting ions in the immediate vicinity of the electrode. Exceeding this value lowers the local nickel-ion concentration at the cathode, leading to diminished efficiency and poorer deposit quality. The dependence of the limiting current density *I_lim_* [A·m^−2^] on mixing intensity can be expressed as

(1) $$I_{lim}=z\cdot F\cdot k\cdot c$$

where:

*z* is the number of electrons transferred per nickel ion [-],

*F* is Faraday’s constant [C·mol^−1^],

*k* is the mass-transfer coefficient (related to stirring rate and diffusion) [m·s^−1^], typical values for electrowinning with mechanical mixer is k_m_ ≈ 1 × 10^−4^ − 5 × 10^−3^ m/s

*c* is the ion concentration in the solution [mol·m^−3^].

The larger the k value (i.e., the more vigorous the mixing), the higher the limiting current density and the later diffusion limitations set in Ref. [24].

It can be concluded that the optimal current density for nickel electrolysis is not a fixed value—it depends on the nickel concentration in the solution and the mass-transfer coefficient. Scientific papers often report an optimal range of 2–2.5 A dm^−2^ by nickel concentration 40–60 g·L^−1^ [20,25,26].

The current density is 2–6 A dm^−2^ [25,27]; higher values speed up deposition but may impair uniformity and promote dendrite growth. Current efficiencies of 90–95% are attainable when bath composition, temperature, agitation intensity and the use of additives (both organic and inorganic) are properly optimized [26]. Final parameter selection must take into account the specifics of the recycling process, including impurity levels, the presence of other metals in solution and the required nickel purity.

In electrolytic nickel deposition, solution agitation plays a key role in determining deposit quality, as it affects mass-transport conditions and maintains stable electrochemical parameters near the electrode surface. More vigorous mixing thins the diffusion boundary layer, ensuring an even supply of Ni^2+^ ions to the cathode and preventing local electrolyte depletion. The resulting reduction in diffusion-layer thickness leads to a more uniform deposit morphology and less susceptibility to micropores or inclusions caused by uncontrolled nucleation. Agitation also limits local pH gradients and the build-up of hydrogen bubbles at the cathode surface, producing smoother, better-adhering deposits [3].

The aim of this study is to evaluate the technological feasibility of nickel electrowinning directly from real hydrometallurgical leachates generated during recycling of lithium-ion battery black mass. Rather than performing a classical design-of-experiments, this study adopts a sequential process optimization strategy typical for scaling up hydrometallurgical technologies, rather than a fundamental mechanistic study of isolated parameters. The recovered material should display at least technical-grade purity (>90%), a uniform structure (free of burrs that could cause short circuits) and good adhesion to the cathode. Main goal the ability to build up a thick metallic layer over extended deposition times.

The article compares two electrolysis concepts: (i) a batch system in which the Ni^2+^ concentration steadily declines as the reaction proceeds, and (ii) a semi-continuous variant in which a constant nickel concentration is maintained by passing the electrolyte through a bed of Ni(OH)_2_ and analyzes the interrelationship between optimum current density, nickel-ion concentration, and diffusion coefficient (mixing intensity), as well as the influence of additives such as PEG-4000, borate, and sodium sulfate on deposit quality.

Two working hypotheses were formulated:

**H1.**  *In a batch electrowinning mode with decreasing Ni^2+^ concentration, proportionally lowering the applied current density should enable continuous nickel deposition at technical-grade quality without requiring a separate Ni(OH)_2_ precipitation and washing step, thereby reducing water consumption and flowsheet complexity.*

**H2.**  *Stabilizing the Ni^2+^ concentration through controlled dissolution of Ni(OH)_2_ should increase process robustness, allow operation at higher and more stable current densities, and yield thicker and more uniform metallic nickel deposits.*

The scope of the study is therefore to compare these two technologically plausible process concepts, assess their operational stability, identify the dominant limiting factors, and evaluate whether thick (>0.4 mm), uniform metallic nickel can be produced from real leach solutions despite natural compositional fluctuations.

The literature already describes in detail how individual electrolysis parameters—such as current density, temperature, pH, agitation, nickel concentration and organic/inorganic additives—affect nickel deposition from synthetic sulfate electrolytes [20,25,28]. These studies provide well-established trends (e.g., improved morphology with increasing temperature, diffusion limitations at low Ni^2+^ concentration, or stress-modifying effects of additives). Reproducing these parameter-by-parameter relationships was therefore not the objective of the present work.

Instead, the purpose of this study is to evaluate the combined, practical effect of these variables under realistic hydrometallurgical conditions, where electrolyte composition is inherently variable and multiple parameters interact simultaneously. In such industrially relevant environments, the key question is not how each variable behaves in isolation—which is well understood—but whether stable, thick and coherent metallic nickel can be deposited from actual recycling leachates.

## 2. Materials and Methods

The feedstock used in this study was a sulfate-based metal solution obtained by acid leaching of battery black mass followed by a hydrometallurgical treatment sequence. That sequence comprised the removal of manganese, copper, iron and aluminum [29], and subsequent solvent extraction of cobalt.

The solution destined for the nickel-electrolysis experiments was characterized by atomic-absorption spectroscopy (AAS) with a Perkin Elmer PinAAcle 350 (Waltham, MA, USA).

It should be noted that a natural consequence of changing the technological variant is a different concentration of sodium and lithium in the feed material (Table 1). The precipitated nickel hydroxide must be washed before being dissolved in the electroplating bath. Depending on the degree to which the material is washed, lithium and sodium will be present in the electroplating bath (Table 2). The concentration of cobalt depends on the effectiveness of the methods used to separate nickel and cobalt, for example, by solvent extraction.

The experimental setup consisted of a 20 dm^3^ electroplating tank fitted with a heating jacket. The bath was further agitated by a mechanical stirrer and equipped with probes for pH and conductivity monitoring. One cathode and two anodes, all measuring 200 × 100 mm and spaced 40 mm apart, were connected to a power supply. In the operating mode with a falling Ni^2+^ concentration, the electrolyte was recirculated from the plating bath through a 30 dm^3^ buffer tank to slow the rate of concentration drop. The solution was alkalized with 2 M sodium hydroxide delivered by a dosing pump linked to the pH meter, enabling precise pH control; more concentrated NaOH could create local supersaturation and precipitate nickel hydroxide in the bath. In the variant using nickel-hydroxide dissolution, two parallel capsules packed with Ni(OH)_2_ were installed in the circulation loop. Each capsule was 210 mm long and had internal diameter of 45 mm, resulting in a cross-sectional area of about 15.9 cm^2^, and finished with a porous bottom on both ends. Total volume of each capsule was approx. 0.34 dm^3^, although only 80% were utilized to maintain free headspace over Ni(OH)_2_ bed. Bath was fed via peristaltic pump to capsules from top to bottom to ensure all solids maintain within capsules. The dissolution rate was naturally controlled by the acidity generated at the anode; balancing the pH stabilized the Ni concentration automatically.

The process was carried out at 60 °C. Elevated temperature favored the formation of higher-quality deposits—the coatings flaked off far less.

AISI 316 stainless steel was chosen as the cathode material because the metallic deposits can easily be stripped from its surface. In the fundamental trials, mixed-metal-oxide (MMO) anodes produced by Shaanxi Yunzhong metal technology Co., Ltd. (Baoji, China) were used, consisting of titanium coated with ruthenium- and iridium-oxide layers (RuO_2_, IrO_2_). This design was selected mainly for its high corrosion resistance and dimensional stability. RuO_2_ and IrO_2_ also exhibit high electrochemical activity, reducing the overall energy consumption of electrolysis compared with higher-overpotential anodes (e.g., lead).

Electrolysis was performed under galvanostatic conditions. In Experiment A, the nickel-ion concentration in the electrolyte decreased over time, whereas in Experiment B it was kept constant. The experimental variables were current density, mixing intensity and the effect of additives.

The cathode surface was prepared by degreasing and then immersing it for 24 h in a 2 M boric acid solution. Collected deposits were dried.

The electrolytic recovery of nickel was carried out in two modes:

a. Directly from the process extract (Series A)

In this approach, the nickel concentration in the electrolyte steadily declined, and the solution was alkalized with sodium hydroxide—pH control between experiment was quite stable (between 4.07 and 4.45). The use of additives was restricted because the extract was intended for further downstream processing.

When electrolysis is run with a falling Ni^2+^ concentration, electrolyte agitation plays a crucial role through its influence on the mass-transfer coefficient k. As the nickel content in solution drops, ion transport to the cathode surface becomes progressively more difficult and the reaction turns diffusion-controlled. To counteract this phenomenon, the mixing intensity between experiments was increased. Insufficient stirring produces a low-velocity boundary layer that further limits the supply of Ni(II) ions.

b. Dissolution of nickel hydroxide in the electrowinning bath (Series B)

In this operating mode, an electrowinning bath is set up by dissolving nickel hydroxide in a sulfuric-acid solution. The Ni(OH)_2_ itself is first precipitated from the process extract via alkalization, washed and is then used to neutralize the bath and keep its nickel concentration constant. Because the electrowinning bath is isolated from the main process stream, the operation can be run continuously, provided the bath is continuously replenished with nickel hydroxide. This strategy makes it possible to produce a bath of any required nickel concentration, while the use of organic additives in the bath has no impact on the downstream processing steps. In series B, only the results obtained with the use of bath additives were presented. Control experiments conducted without additives produced results similar to those of series A and therefore, were not discussed in detail here. The use of additives was clearly assessed as beneficial in the work Effect of additives on nickel electrowinning, and the concentration values adopted in the present trials were selected based on the results presented therein.

## 3. Results and Discussion

### 3.1. Series A

In series A, the process was run on an electrolyte whose Ni(II) content fell steadily from 49.7 g L^−1^ at the outset (stage A1) to 25 g L^−1^ at the end (stage A8). To counter the growing diffusion limitations, the stirring rate was gradually increased from 300 rpm to 650 rpm. Simultaneously, metered addition of NaOH raised was the pH from ≈3.2 to a maximum of 4.45, limiting electrode corrosion and boosting the solution’s conductivity. As the nickel concentration declined, the current density was reduced from 240 to 65 A m^−2^. From batch A7 onward, 4 g L^−1^ PEG-4000 was added to the bath to improve adhesion and lessen deposit flaking. In experiment A8, the current density was deliberately increased as the primary variable. This adjustment led to a lower-quality deposit, which in turn shortened the overall process duration.

The use of additives in series A was deliberately limited because the post-electrolysis liquor was destined for further processing—lithium removal and sodium-sulfate crystallization. Introducing more organic agents would complicate the downstream steps and add contaminants to the process liquids.

**Figure 2:**
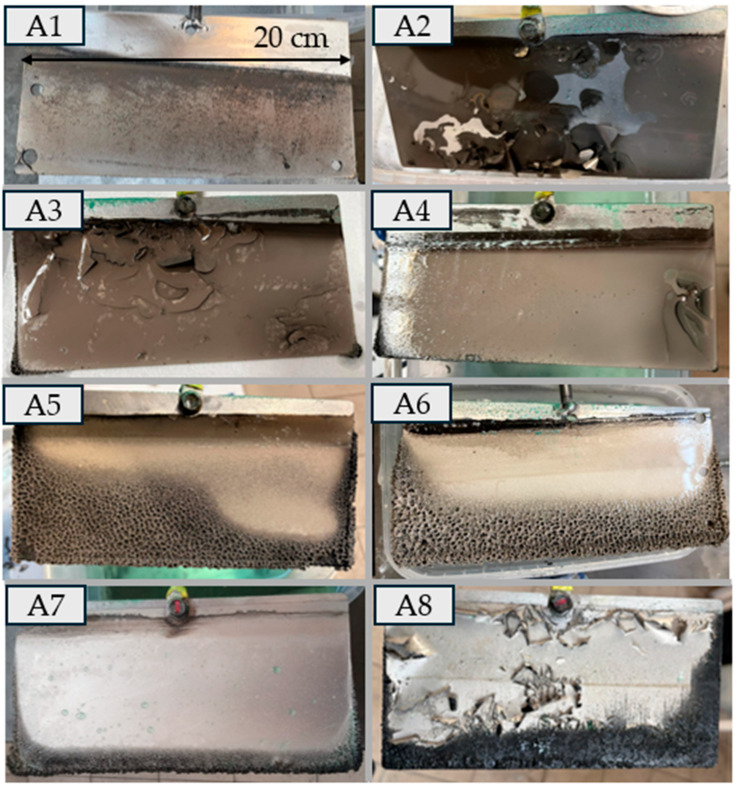
Visual appearance of nickel deposits obtained in Experiment A. The images show the morphology, surface quality, and macroscopic features of the coatings produced under different operating conditions.

In experiment A1, no additives were used. The initial nickel concentration was optimal, and the current density was relatively high (Table 3). The resulting deposit was of fairly good quality—smooth and uniform (Figure 2). However, a “flake” emerged from its surface, leading to contact between the anode and cathode.

**Figure 3:**
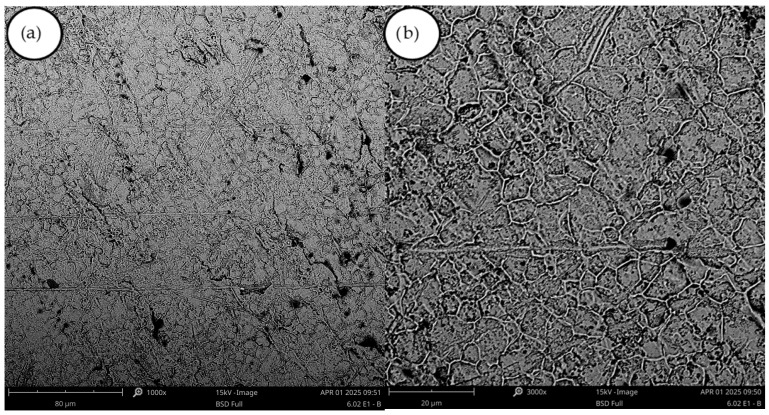
SEM images of the deposit obtained in experiment (A2): (**a**) magnification 1000×, (**b**) magnification 3000×.

In experiment A2, the current density was reduced from 240 to 160 A dm^−2^, resulting in a significant deterioration in coating quality (Table 3). Although the sheet was smooth in many areas, flaking in the form of circular scales was observed in the central region of the cathode (Figure 2). In the SEM image (Figure 3), the deposit appeared to be the smoothest among all the deposits examined; however, it did not meet the other experimental criteria.

In experiments (A3), (A4), and (A5), the current density was gradually increased to fall within the range of 160 to 250 A/dm^2^ (Table 3). Among these, the sheet from experiment (A4), obtained at a current density of 180 A dm^−2^, exhibited the most favorable visual characteristics. This experiment also yielded a high current efficiency (88%). However, after 5 h, the sheet caused a short circuit due to contact with the anode.

In contrast, experiment (A5) resulted in a significantly longer deposition time (9 h), compared to 1–2 h in experiments (A1) and (A2), and 5 h in (A3) and (A4), representing a notable improvement. Nevertheless, the deposit in (A5) underwent a dramatic change in appearance. In some areas, the sheet was smooth and resembled that from experiment (A4), but in the lower part of the cathode, the structure became porous (Figure 2).

**Figure 4:**
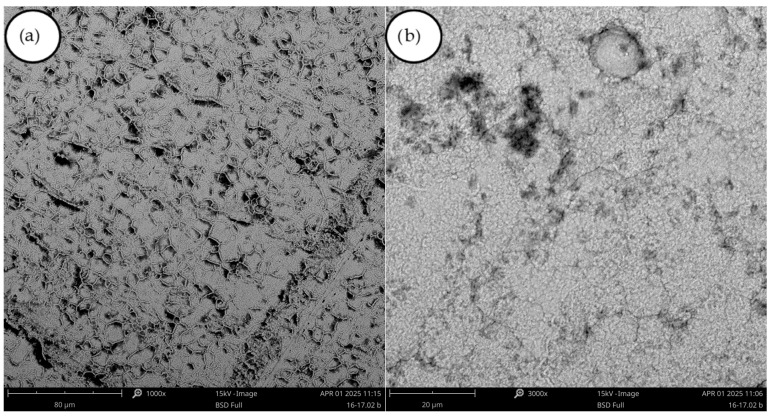
(**a**) SEM images of the deposit obtained in experiment A5, magnification 1000×, (**b**) SEM images of the deposit obtained in experiment A8 (bright place), magnification 3000×.

In experiment (A6), the current density was reduced twofold. The resulting deposit did not differ significantly from that obtained in experiment (A5). The visual changes observed in deposits from experiments (A5), (A6), (A7), and (A8) suggest a substantial influence of PEG-4000 additive on surface morphology. The material transitioned from a porous structure to a smooth and glossy finish with black powder on edges. However, in the SEM images of the samples, increased porosity is visible in experiments (A5) and (A8), compared to experiment (A2) (Figure 3 and Figure 4).

### 3.2. Series B

In series B, only the results obtained with the use of bath additives were presented. Control experiments conducted without additives produced results similar to those of series A and therefore, were not discussed in detail here. The use of additives was clearly assessed as beneficial in the work Effect of additives on nickel electrowinning, and the concentration values adopted in the present trials were selected based on the results presented therein.

**Figure 5:**
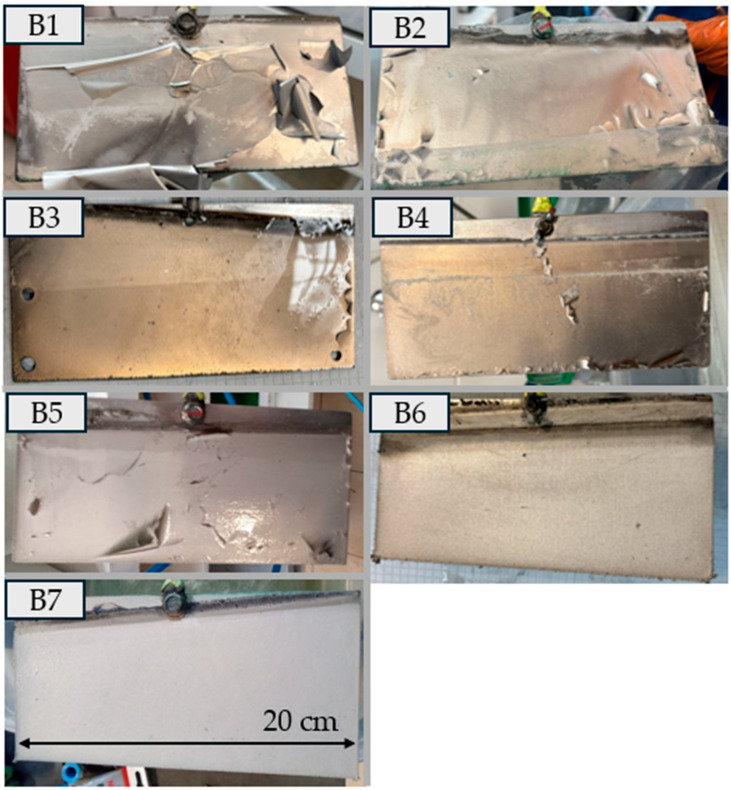
Visual appearance of nickel deposits obtained in Experiment B. The images show the morphology, surface quality, and macroscopic features of the coatings produced under different operating conditions.

**Figure 6:**
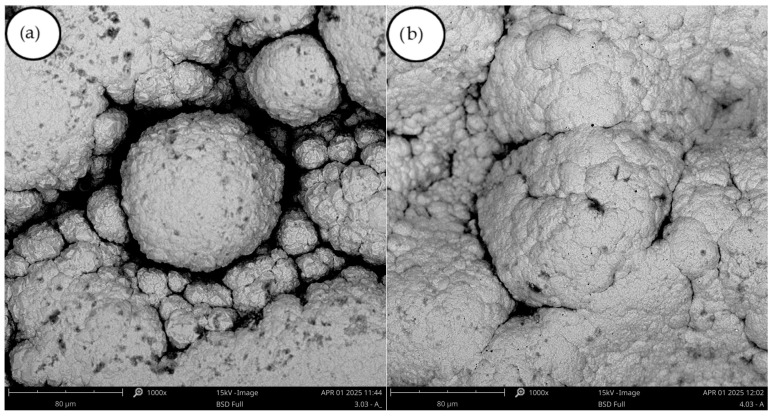
(**a**) SEM images of the deposit obtained in experiment B6, magnification 1000×; (**b**) SEM images of the deposit obtained in experiment B7, magnification 1000×.

In experiment (B1), the deposit obtained was a highly flaking film (Figure 5). Its structure was smooth and shiny. In experiments (B2) and (B3), reducing the current density decreased the flaking. In experiments (B3) and (B4) PEG-4000 was added (Table 4). The deposit became more yellow, which is completely different from its behavior in experiments (A7)–(A8) (Figure 3), where the addition of PEG-4000 caused the deposit to become significantly lighter (Figure 5). In experiment (B5), the boric acid content was increased, while in experiments (B6) and (B7) the sodium sulfate concentration was doubled. The effect on the deposit was significant—the deposit became much lighter in color and showed no tendency to crack or flake. Control tests were carried out, and no differences were observed between the deposits obtained. The thickest of them was removed in a single piece. The deposit thickness was measured at 0.4 mm. The metal was fairly hard yet flexible, and it clearly met the initial requirements set for the experiment. The SEM images of (B6) and (B7) (Figure 6) show a “cauliflower-like” or “clustered” structure, in contrast to the smooth surface obtained in experiment (A2). It is possible that the higher smoothness is associated with greater internal stresses, which in turn result in cracking of the deposit.

Process durations varied between experiments. In runs (A1)–(A4), (A8) and (B1)–(B5), a metallic deposit grew from the cathode to the anode, causing a short circuit within the defined process time. For the remaining runs the minimum process duration was set at 8 h (Table 6). That interval appears sufficient to keep industrial cathode changes manageable, and the deposit can then be removed from the cathode in one piece.

In the EDS results (Table 5) for both experimental series, a relatively high nickel content is obtained. The samples in series (A6) and (A7) may not have been cleaned thoroughly enough, hence the increased sodium and sulfur content. The cobalt content in the samples can be considered acceptable. The presence of oxygen may be due to surface oxidation of the samples.

To complement the SEM-EDS measurements, sample (B7) was additionally analyzed using X-ray fluorescence spectroscopy (XRF) on a Bruker S8 TIGER spectrometer (Billerica, MA, USA). The analysis was performed in the QuantExpress (standardless) mode using the FullAnalysis algorithm. The obtained composition is dominated by Ni (93.4 wt%), with minor concentrations of Co (2.34 wt%), Fe (2.1 wt%) and Cr (1.05 wt%), and trace levels of several additional elements (Na, S, Si, Mg, P, Zn, Cl and Mo).

A comparison with the SEM-EDS (Table 5) results shows very good consistency for the minor alloying elements Co, Fe and Cr, whose concentrations fall within the expected uncertainty range of both techniques. The apparent discrepancy in Ni content (XRF: 93.4 wt% vs. EDS: ~86–87 wt%) can be attributed to the intrinsic limitations of EDS quantification. SEM-EDS measurements were performed on the near-surface region, which is partially oxidized, as evidenced by the high oxygen content detected in the EDS spectra (27–33 at%). This local surface oxidation leads to an artificial reduction in the apparent Ni concentration and is a well-documented effect in EDS analysis of Ni-rich materials. In contrast, XRF probes the bulk composition of the entire sample and is largely insensitive to surface oxidation, thus providing a more accurate representation of the true alloy matrix.

Furthermore, XRF detected several light and trace elements (e.g., Na, Si, Mg, P, Zn, Cl, Mo) at concentrations between 100 and 800 ppm. These species were either below the detection limit of SEM-EDS or present at levels indistinguishable from spectral background, which is typical for EDS due to its limited sensitivity to low-Z and low-concentration elements.

Overall, the combined results confirm that the bulk composition of sample (B7) is dominated by Ni with minor additions of Co, Fe and Cr, while the higher oxygen content and slightly reduced Ni concentration observed in SEM-EDS reflect only the chemical state of the near-surface region and not the composition of the underlying alloy.

## 4. Conclusions

By comparing electrowinning under decreasing Ni^2+^ concentration (Mode A) with a semi-continuous stabilization strategy based on controlled dissolution of Ni(OH)_2_ (Mode B). we demonstrate that:

1. In Experiment B, robust metallic sheets with a thickness of 0.4–0.5 mm and high purity (>90%) were obtained.
2. The method is compatible with existing hydrometallurgical plants, since Ni(OH)_2_ used to stabilize the bath can be precipitated directly from the same leachate stream.

Therefore, while current recycling technologies recover nickel mostly as a chemical compound, the approach presented in this work enables the integration of nickel electrowinning as a viable end-step, generating a metallic product suitable for direct reuse or refining.

For efficient nickel electrowinning, it is advantageous to maintain a constant nickel concentration in the electrolyte (variant B). Although stabilizing the Ni^2+^ concentration is an advantageous condition for process continuity, it was not sufficient on its own to prevent deposit defects; high-quality, adherent metallic sheets were achieved only when concentration control was synergistically combined with the use of additives H_3_BO_3_, Na_2_SO_4_, PEG-4000). Effects of additives on the morphology and properties of the deposited nickel have been extensively described in the scientific literature and were also confirmed in the present study.

Current (Faradaic) efficiency proved to be the primary determinant of energy intensity (kWh kg^−1^) in our nickel electrowinning tests (Table 6). Because the deposited mass is proportional to charge × efficiency while energy is proportional to cell voltage × charge, the specific energy consumption scales approximately as V/CE. In the A-series, where bath Ni^2+^ concentration slowly depleted during runs, CE fell and energy intensity rose (typical kWh kg^−1^ in the A-series: 2.49–4.39, with outliers up to ~4.75). In contrast, the B-series—run with a stabilized Ni concentration—achieved consistently higher CE (up to 93%) and lower energy consumption (as low as ~2.84 kWh kg^−1^) even at high current densities (~190 A m^−2^). These results indicate that maintaining ionic supply to the cathode (continuous or frequent make-up of Ni^2+^) is more important than lowering current density per se: with sufficient mass transport, high current density is compatible with high Faradaic efficiency and low energy intensity.

On the other hand, direct electrolytic removal of nickel from the process solution—as in variant A—undoubtedly introduces additional operational and control challenges. Nevertheless, some of the obtained deposits (A1, A4, A7) are not dramatically poor, indicating that the development and implementation of such a technology is not necessarily doomed to failure from the outset. However, it would certainly require a more demanding level of process stabilization, phase purity control, and management of electrochemical parameters.

Controlling pH between 3.0 and 4.5 was critical: overly acidic media promoted hydrogen evolution, whereas higher pH caused precipitation of metal hydroxides, complicating electrolyte management. Mode A displayed a broad efficiency range (49–88%), partly due to difficulties detaching flaking deposits. In Mode B, final trials (B6) and (B7) achieved 88–90% at ~200 A m^−2^, a very good result. Stabilising Ni(II) concentration, providing adequate agitation, and carefully setting pH, temperature and additive levels enabled high-quality nickel deposits and higher current efficiency. Processes run with a falling Ni concentration gave more variable deposit quality, underscoring the importance of mass-transport control and bath optimisation when electrowinning nickel from sulphate solutions. Variant B should be considered the recommended approach for industrial implementation.

## Figures and Tables

**Table 1:** Initial and final composition of the electroplating bath in Experiment A. The table summarizes the concentrations of key metal ions and additives before and after the electroplating process, illustrating how the bath chemistry changed as a result of metal deposition.

	Concentration [mg dm^−3^]							
	Li	Co	Ni	Al	Mn	Cu	Fe	Na
**Initial composition of solution (A1)**	4238	717	49,700	30	99	1.3	0.7	12,440
**Final composition of solution (A8)**	4115	85	25,000	5.4	87	<LOQ	<LOQ	12,118

**Table 2:** Initial and final composition of the electroplating bath in Experiment B. The table summarizes the concentrations of key metal ions before and after the electroplating process, illustrating how the bath chemistry changed as a result of metal deposition.

	Concentration [mg dm^−3^]							
	Li	Co	Ni	Al	Mn	Cu	Fe	Na
**Initial composition of solution (B1)**	553	496	37,200	16	71	0.96	0.58	6470
**Final composition of solution (B7)**	708	22	39,500	5.9	53	<LOQ	<LOQ	12,920

**Table 3:** Operating conditions applied in Experiment A. The table summarizes the process parameters used in each sub-experiment (A1–A8), including average bath pH, initial nickel concentration, concentration of additives, applied current density, and stirring speed.

Experiment	A1	A2	A3	A4	A5	A6	A7	A8
Average pH	4.23	4.38	4.10	4.26	4.45	4.17	4.07	4.12
Initial Ni concentration [g/L]	49.7	44.8	39.2	35.1	31.8	29.9	28.2	25.0
PEG 4000 concentration [g/L]	0.0	0.0	0.0	0.0	0.0	0.0	4.0	4.0
Current density (A/m^2^)	240	160	176	181	196	91	65	117
Stirring speed [rpm]	300	300	530	650	650	650	650	650

**Table 4:** Operating conditions applied in Experiment B. The table summarizes the process parameters used in each sub-experiment (B1–B7), including average bath pH, initial nickel concentration, concentration of additives, applied current density, and stirring speed.

Experiment	B1	B2	B3	B4	B5	B6	B7
Average pH	4.04	4.14	4.40	4.29	4.08	4.38	4.22
Initial Ni^2+^ concentration [g/L]	37.2	39.7	38.3	37.5	35.6	38.1	39.5
PEG 4000 concentration [g/L]	0.0	0.0	4.0	4.0	4.0	4.0	4.0
H_3_BO_3_ concentration [g/L]	6.2	6.2	6.2	6.2	15.0	15.0	15.0
Na^+^ concentration * [g/L]	8.4	8.4	8.4	8.4	8.4	10.0	22.0
Current density (A/m^2^)	297	232	198	203	203	202	193
Stirring speed [rpm]	650	650	650	900	900	900	900

* added as Na_2_SO_4._

**Table 5:** Averaged EDS analysis results for selected samples.

Sample	Ni	Co	O	Na	S	Fe
**A2**	75.01 ± 17.31	10.90 ± 6.33	12.76 ± 15.28	LOD	3.90 ± 5.81	LOD
**A6**	87.57 ± 2.95	3.41 ± 0.43	7.83 ± 3.50	LOD	0.45 ± 0.12	LOD
**A7**	86.21 ± 5.99	2.49 ± 0.68	7.52 ± 1.44	6.21 ± 1.30	1.34 ± 0.28	LOD
**B4**	89.68 ± 1.31	3.56 ± 1.22	5.13 ± 2.92	LOD	0.72 ± 0.11	2.25 ± 0.58
**B6**	90.34 ± 0.75	3.23 ± 0.59	6.44 ± 0.35	LOD	LOD	LOD
**B7**	86.81 ± 2.32	2.19 ± 0.99	10.24 ± 1.20	LOD	1.01 ± 0.24	1.54 ± 0.67

**Table 6:** Experimental Conditions and Performance Outcomes for A- and B-Series Electrochemical Deposition Tests, Including Current Efficiency, Operating Time, Cell Parameters, Deposit Mass, and Energy Consumption.

Experiment	CE [%]	Time [h]	Avg I (A)	Avg V (V)	j (A·m^−2^)	Deposit Mass ± Δm (g)	E ± ΔE (Wh)	kWh/kg ± Δk
A1	76	1.8	8.98	3.1	236.3	13.6 ± 0.1	50.7 ± 0.2	3.725 ± 0.030
A2	59	1.2	5.73	2.8	150.9	4.3 ± 0.1	18.5 ± 0.1	4.334 ± 0.103
A3	78	5.0	4.54	2.63	119.4	19.5 ± 0.1	60.1 ± 0.3	3.079 ± 0.021
A4	88	5.1	7.15	2.98	188.2	34.9 ± 0.1	108.0 ± 0.4	3.093 ± 0.014
A5	67	9.0	7.28	2.89	191.5	48.1 ± 0.1	189.4 ± 0.7	3.939 ± 0.017
A6	60	17.7	7.32	2.89	192.8	85.0 ± 0.1	373.8 ± 1.4	4.399 ± 0.017
A7	86	10.5	2.35	2.35	61.6	23.3 ± 0.1	58.1 ± 0.4	2.496 ± 0.018
A8	49	4.3	4.22	2.55	111.1	9.8 ± 0.1	46.5 ± 0.2	4.753 ± 0.053
B1	46	3.8	10.66	2.87	280.6	20.4 ± 0.1	116.3 ± 0.4	5.699 ± 0.035
B2	52	3.9	3.94	2.51	103.6	8.8 ± 0.1	38.9 ± 0.2	4.408 ± 0.054
B3	63	4.3	5.92	2.99	155.7	17.4 ± 0.1	75.6 ± 0.3	4.334 ± 0.030
B4	78	5.4	7.14	2.96	188	32.6 ± 0.1	113.1 ± 0.4	3.466 ± 0.017
B5	93	5.6	7.28	2.89	191.6	41.3 ± 0.1	117.2 ± 0.4	2.838 ± 0.013
B6	88	13.6	7.29	2.92	191.8	95.5 ± 0.1	289.5 ± 1.1	3.030 ± 0.012
B7	90	8.2	7.36	2.84	193.7	59.6 ± 0.1	171.8 ± 0.6	2.882 ± 0.012

## Data Availability

The original contributions presented in this study are included in the article. Further inquiries can be directed to the corresponding author.
